# Extracellular Vesicles From Senescent Cancer Cells Are Necessary and Sufficient to Drive Paracrine Senescence

**DOI:** 10.1111/acel.70722

**Published:** 2026-09-23

**Authors:** Valentin Estevez‐Souto, Alex Miralles‐Dominguez, Pablo Pedrosa, Patricia Lado‐Fernandez, Miguel A. Prados, Alejandro Failde‐Fiestras, Raquel Paredes‐Paredes, Jorge Ruz‐Ortega, Maria J. Alonso, Martina Migliavacca, Ester Polo, Rebeca Alvarez‐Velez, Enrique Vazquez de Luis, Ana Dopazo, Gabriela N. Condezo, Carmen San Martin, Miguel Gonzalez‐Barcia, Ramón Lobato‐Busto, Pilar Ximénez‐Embún, Javier Muñoz, Manuel Collado, Sabela Da Silva‐Alvarez

**Affiliations:** ^1^ Laboratory of Cell Senescence, Cancer and Aging, Center for Research in Molecular Medicine and Chronic Diseases (CiMUS) University of Santiago de Compostela, Health Research Institute of Santiago de Compostela (IDIS) Santiago de Compostela Spain; ^2^ Laboratory of Nanomedicine and Drug Delivery, Center for Research in Molecular Medicine and Chronic Diseases (CiMUS) University of Santiago de Compostela, Health Research Institute of Santiago de Compostela (IDIS) Santiago de Compostela Spain; ^3^ Department of Pharmacy and Pharmaceutical Technology, School of Pharmacy University of Santiago de Compostela Santiago de Compostela Spain; ^4^ Instituto Madrileño de Estudios Avanzados en Nanociencia (IMDEA Nanociencia) Madrid Spain; ^5^ Department of Inorganic Chemistry, Center for Research in Biological Chemistry and Molecular Materials (CiQUS) University of Santiago de Compostela Santiago de Compostela Spain; ^6^ Department of Biochemistry and Molecular Biology, Center for Research in Biological Chemistry and Molecular Materials (CiQUS) University of Santiago de Compostela Santiago de Compostela Spain; ^7^ Genomics Unit Centro Nacional de Investigaciones Cardiovasculares Madrid Spain; ^8^ Department of Macromolecular Structures Centro Nacional de Biotecnología (CNB‐CSIC) Madrid Spain; ^9^ FarmaChusLab Group Health Research Institute of Santiago de Compostela (IDIS) Santiago de Compostela Spain; ^10^ Hospital Pharmacy Department University Hospital Complex of Santiago de Compostela (CHUS) Santiago de Compostela Spain; ^11^ Department of Medical Physics Hospital Clínico Universitario de Santiago de Compostela, Servizo Galego de Saúde (SERGAS) Santiago de Compostela Spain; ^12^ Proteomics Unit Spanish National Cancer Research Centre (CNIO) Madrid Spain; ^13^ Cell Signalling and Clinical Proteomics Group Biobizkaia Health Research Institute Barakaldo Spain; ^14^ Ikerbasque Basque Foundation for Science Bilbao Spain; ^15^ Department of Immunology and Oncology (DIO) Centro Nacional de Biotecnología (CNB‐CSIC) Madrid Spain

**Keywords:** cellular senescence, extracellular vesicles, paracrine senescence, SASP

## Abstract

Cellular senescence exerts powerful non‐cell autonomous effects through the senescence‐associated secretory phenotype (SASP). This SASP comprises soluble factors and extracellular vesicles (EVs). Although soluble SASP components can induce senescence in neighboring cells, the specific contribution of EVs to paracrine senescence is poorly defined. Here, we show that EVs released by senescent tumor cells are necessary and sufficient to propagate senescence. Conditioned media from bleomycin‐induced senescent A549 cells triggered a growth arrest with morphological changes and upregulation of senescence markers in recipient tumor cells. Pharmacological inhibition of EV biogenesis using GW4869 or genetic downregulation of the EV secretion mediator RAB27A markedly attenuates paracrine senescence without affecting soluble SASP factor secretion or the senescent state of producer cells. Proteomic characterization reveals that senescent EVs exhibit a distinct molecular signature enriched for extracellular components and processes related to wound healing and hemostasis. Importantly, purified senescent EVs, devoid of soluble SASP factors, fully recapitulated paracrine senescence induction. These findings identify senescent EVs as key autonomous SASP effectors and highlight vesicular pathways as potential therapeutic targets in cancer and therapy‐induced senescence.

## Graphical abstract

Senescent cancer cells exhibit a secretory phenotype, known as SASP, capable of inducing paracrine senescence. However, the main factors within the SASP triggering this process are unknown. Here, we demonstrate that EVs within the SASP are necessary and sufficient to induce paracrine senescence.

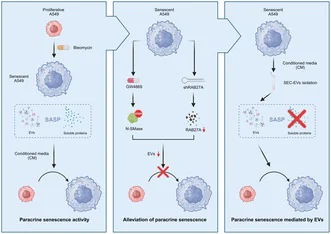

## 1 Introduction

Cellular senescence, a state of stable cell cycle arrest, represents a fundamental tumor suppressive mechanism that prevents the proliferation of damaged or oncogene‐activated cells (Collado et al. 2007; Serrano 2011). Paradoxically, senescent cells can persist in tissues and secrete a complex repertoire of factors collectively termed the senescence‐associated secretory phenotype (SASP), which can exert pro‐tumorigenic effects through the remodeling of the microenvironment (Acosta et al. 2013; Coppé et al. 2010). The SASP comprises a complex mixture of soluble factors such as cytokines, chemokines, growth factors, and extracellular matrix remodelers with functional consequences that vary depending on the context and that might result in beneficial or detrimental effects. Recently, it has become apparent that apart from this soluble SASP there is also a vesicular SASP, formed mainly by extracellular vesicles (EVs). Potentially, both the soluble and the vesicular fraction can contribute to the heterogeneous functional outcomes of senescence in cancer progression promoted by the SASP (Borghesan et al. 2019).

While the soluble fraction of the SASP has been extensively characterized for its capacity to induce senescence in neighboring cells, a phenomenon termed paracrine senescence, the specific contribution of EVs to this process remains incompletely understood. EVs serve as critical mediators of intercellular communication by transferring bioactive cargo, including proteins, lipids, and nucleic acids, between cells (Raposo and Stoorvogel 2013; Welsh et al. 2024). Recent studies have revealed that senescent cells release increased quantities of EVs, which have been implicated in diverse biological processes ranging from immune modulation to tissue repair (Estévez‐Souto et al. 2023; Takasugi 2018). However, whether senescent EVs possess autonomous paracrine senescence‐inducing activity, distinct from soluble SASP components, has not been rigorously tested.

The functional dissection of SASP components carries significant therapeutic implications. Pharmacological strategies targeting senescent cells using compounds that induce specific cytotoxicity, known as senolytics, or that dampen down their secretory output, known as senomorphics, are actively being developed for cancer and age‐related pathologies (Hickson et al. 2019). Understanding whether EVs represent a dispensable byproduct or an essential effector of paracrine senescence could inform the design of more precise interventions. Moreover, given that EVs are membrane‐bound and potentially druggable through distinct mechanisms compared to soluble factors, identifying their specific contributions may reveal new therapeutic vulnerabilities.

In this study, we systematically investigate the role of EVs secreted by senescent tumor cells in mediating paracrine senescence. Using bleomycin‐induced senescent A549 lung adenocarcinoma cells as a model, we demonstrate that conditioned medium from senescent cells induces senescence markers in recipient tumor cells, including altered morphology, senescence‐associated β‐galactosidase (SA‐β‐GAL) activity, and upregulation of the cyclin‐dependent kinase inhibitor *CDKN1A*. Through both pharmacological inhibition of EV biogenesis using GW4869 and genetic downregulation of the EV secretion mediator *RAB27A*, we establish that reduction of EV release markedly attenuates paracrine senescence without affecting soluble SASP factor secretion. Proteomic characterization of senescent EVs reveals distinct molecular signatures consistent with previously reported senescent vesicle profiles. Critically, EVs isolated by size‐exclusion chromatography retain full paracrine senescence‐inducing capacity in the absence of soluble SASP components. These findings establish senescent EVs as autonomous mediators of paracrine senescence and highlight their potential as specific targets for modulating the senescence secretory response in cancer and aging.

## 2 Results

### 2.1 Conditioned Media From Senescent A549 Cells Induce Paracrine Senescence

**FIGURE 1:**
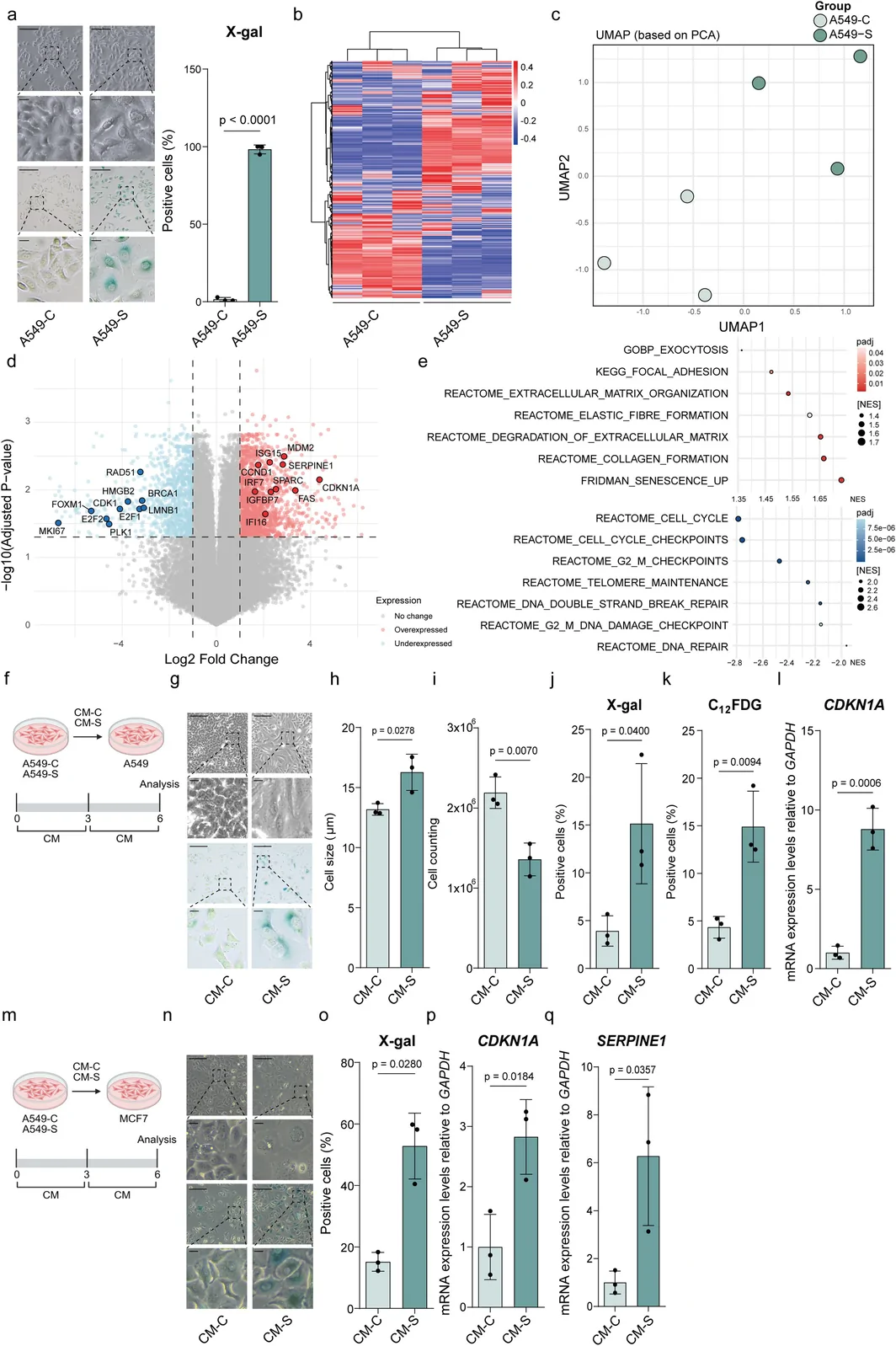
Conditioned media from chemotherapy‐induced senescent A549 cells induce paracrine senescence in proliferative A549 cells. (a) Representative images of cell morphology and SA‐β‐GAL staining of proliferative (A549‐C) and bleomycin‐induced senescent (A549‐S) A549 cells (scale, 200 μm; scale in zoomed area, 20 μm). Quantification of the staining. (b) Heatmap representation of RNA‐seq analysis of A549‐C and A549‐S. (c) UMAP showing group clustering. (d) Volcano plot of differentially expressed genes. (e) Dot plot analysis of differentially expressed pathways. Red dots represent upregulated pathways, and blue dots represent downregulated pathways. (f) Schematic representation of proliferative (CM‐C) and senescent (CM‐S) A549 CM treatment over proliferative A549 cells. (g) Representative images of cell morphology and SA‐β‐GAL staining, large scale, 200 μm, short scale, 20 μm. (h) Cell size measurement. (i) Cell counting. (j) Quantification of SA‐β‐GAL positive cells using X‐gal substrate. (k) Quantification of SA‐β‐GAL positive cells using C_12_FDG substrate. (l) RT‐qPCR analysis of *CDKN1A*. *GAPDH* was used as housekeeping gene. (m) Schematic representation of proliferative (CM‐C) and senescent (CM‐S) A549 CM over proliferative MCF7. (n) Representative images of cell morphology and SA‐β‐GAL staining, large scale, 200 μm, short scale, 20 μm. (o) Quantification of SA‐β‐GAL positive cells using X‐gal substrate. (p) RT‐qPCR analysis of *CDKN1A*. *GAPDH* was used as housekeeping gene. (q) RT‐qPCR analysis of *SERPINE1*. *GAPDH* was used as housekeeping gene. Significance was calculated using Student's *t*‐test showing the exact *p*‐value. Less than 0.05 was considered statistically significant (*n* = 3).

Secreted factors from senescent primary cells, collectively termed senescence‐associated secretory phenotype (SASP), have been previously reported to be capable of inducing senescence in neighboring cells (Acosta et al. 2013; Acosta et al. 2008; Kuilman et al. 2008). To test the activity of secreted factors from senescent tumor cells, we decided to analyze the conditioned media (CM) derived from senescent human lung adenocarcinoma A549 cells. For this, A549 cells were treated with bleomycin, a DNA damaging agent widely used to induce senescence (Aoshiba et al. 2013; Aoshiba et al. 2003). Treated cells exhibited changes in cell morphology, an increase in SA‐β‐GAL activity, a higher expression of *CDKN1A*, and decreased proliferation (Figure 1a and Figure S1a–f). In addition, the transcriptomic analysis of these bleomycin‐treated A549 cells revealed a clear separation of treated and untreated samples, with the treated cells clustering together and sharing a senescence profile. This was characterized by the altered expression of differentially expressed genes typically found during cell senescence, such as the increase in *CDKN1A*, *SERPINE1*, *MDM2*, or *FAS*, or the decrease in *FOXM1*, *CDK1*, *LMNB1* or *PLK1* (Figure 1b–e and Figure S1g–j).

We collected CM from control proliferative (CM‐C) and senescent (CM‐S) A549 cells and treated proliferative A549 cells for two rounds as recipient cells (Figure 1f). These cells exhibited an enlarged and flattened morphology and reached a lower confluency (Figure 1g–i). Since this was suggestive of an induction of cell senescence, we assessed these cell cultures for SA‐β‐GAL activity. Using both X‐Gal and C_12_FDG as substrates, we observed a clear increase in SA‐β‐GAL positive cells after CM‐S treatment (Figure 1g,j,k and Figure S1k). Cells also presented an upregulation of *CDKN1A* expression (Figure 1l), a cell cycle inhibitor typically involved in the proliferative arrest that characterizes cell senescence. This observation was not restricted to A549 cells, but it was also observed when we used the human breast cancer cell line MCF7 (Figure 1m). Cells changed their morphology when they received CM‐S from senescent A549 cells, were positive for SA‐β‐GAL activity and showed increased expression of *CDKN1A* and the SASP factor, *SERPINE1* (Figure 1n–q). Similarly, these observations were also extended to other additional models of cell senescence. CMs and isolated EVs from irradiated A549 or melanoma SK‐MEL‐147 cells showing features of cell senescence (Figure S2a–c) induced paracrine senescence when added to their proliferative counterparts (Figure S2d–i).

Altogether, these results suggest that CM derived from senescent cancer cell lines can induce paracrine senescence in neighboring cells.

### 2.2 Chemical Inhibition of EVs Biogenesis Reduces Paracrine Senescence

**FIGURE 2:**
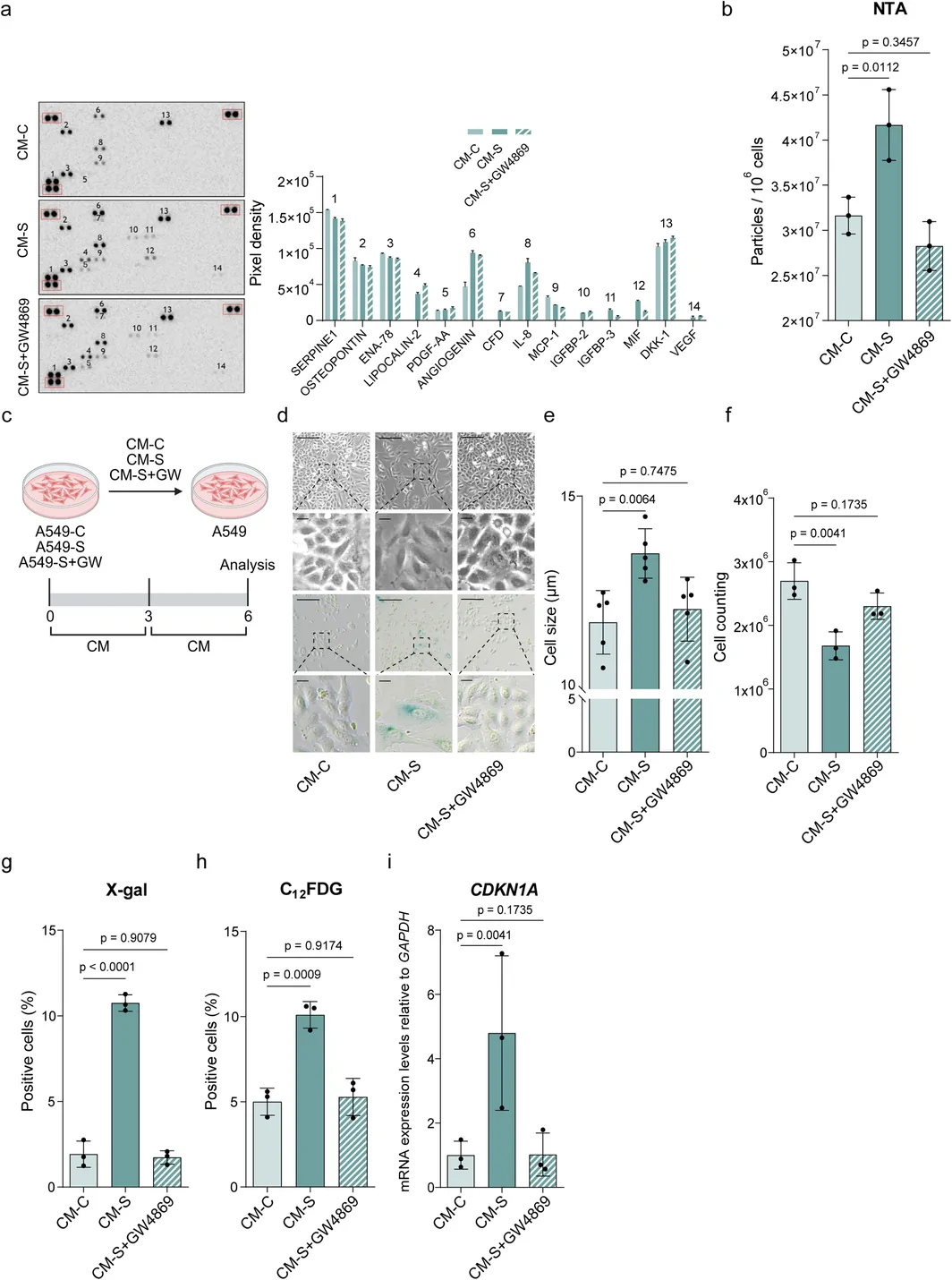
Chemical inhibition of EV biogenesis by GW4869 treatment reduces paracrine senescence. (a) Cytokine array analyses of CMs from proliferative (CM‐C), senescent (CM‐S), and GW4869‐treated senescent cells (CM‐S + GW6849) and their quantification. Red squares mark reference dots. (b) NTA analysis of particles in the CM. (c) Schematic representation of treatments using CMs from proliferative (CM‐C), senescent (CM‐S), and GW4869‐treated senescent cells (CM‐S + GW4869) on proliferative A549. (d) Representative images of morphology and SA‐β‐GAL staining (scale, 200 μm; scale in zoomed area, 20 μm). (e) Cell size measurement. (f) Cell counting. (g) Quantification of SA‐β‐GAL positive cells using X‐gal substrate. (h) Quantification of SA‐β‐GAL positive cells using C_12_FDG substrate. (i) RT‐qPCR analysis of *CDKN1A*. *GAPDH* was used as housekeeping gene. Significance was calculated using one‐way ANOVA showing the exact *p*‐value. Less than 0.05 was considered statistically significant (b–i, *n* = 3; a, *n* = 1).

The SASP is composed of a soluble fraction and extracellular vesicles (EVs). To study the relative contribution of these fractions to the induction of paracrine senescence, we used the chemical inhibitor of EVs biogenesis GW4869. This compound reduces the activity of N‐SMase, an enzyme that catalyzes the synthesis of ceramide, a crucial component of EVs biogenesis (Catalano and O'Driscoll 2020). First, we checked that the treatment with GW4869 did not alter the senescence phenotype of producer cells. Treated cells retained their morphological features and were positive for SA‐β‐GAL staining (Figure S3a–d). Then, we also wanted to confirm that GW4869 was only altering the production of EVs and was not affecting the production or release of soluble factors. For this, we incubated cytokine arrays with CM‐C as a control, and senescent CM derived from senescent A549 cells (CM‐S) or from senescent A549 cells treated with GW4869 to block EVs biogenesis (CM‐S‐GW4869). CM‐S was enriched in several of the cytokines, chemokines and growth factors that have been reported to be part of the SASP compared to CM‐C, as expected. When we incubated the arrays with CM‐S‐GW4869, we observed that this pattern of soluble factors was unaltered by the treatment with GW4869, indicating that it does not modulate the release of the soluble factors of the SASP (Figure 2a). On the other hand, to check the inhibition of EVs biogenesis by the compound, we measured the number of vesicles in the different CMs by nanotracking analysis (NTA). When we analyzed CM‐S, we observed a clear increase in the number of particles detected by NTA compared to CM‐C. This is in agreement with several reports that found an increase in the number of EVs released by senescent cells. In contrast, when we analyzed CM‐S‐GW4869 we observed a drastic reduction in the number of EVs (Figure 2b).

To test the functional consequences of a reduction in the number of EVs in our senescent conditioned media, we treated A549 cells with the different CMs (Figure 2c). Again, CM‐S induced a clear senescent morphology and caused a decreased cell proliferation indicative of paracrine senescence induction. Accordingly, cells stained positive for SA‐β‐GAL activity and showed increased *CDKN1A* expression. In contrast, when cells were treated with CM‐S‐GW4869, containing fewer EVs, cells were morphologically more similar to the ones treated with control CM‐C, were mainly negative for SA‐β‐GAL activity, proliferated as the control cells, and showed lower expression levels of *CDKN1A*. (Figure 2d–i and Figure S3e).

Overall, these data suggest that chemically inhibiting EVs biogenesis by GW4869 leads to a decrease in the number of EVs and an alleviation of paracrine senescence. This effect of GW4869 is not caused by an alteration of the senescent phenotype of the producer cells nor by an alteration of the secretion of soluble factors.

### 2.3 Genetic Downregulation of EVs Secretion Alleviates Paracrine Senescence

**FIGURE 3:**
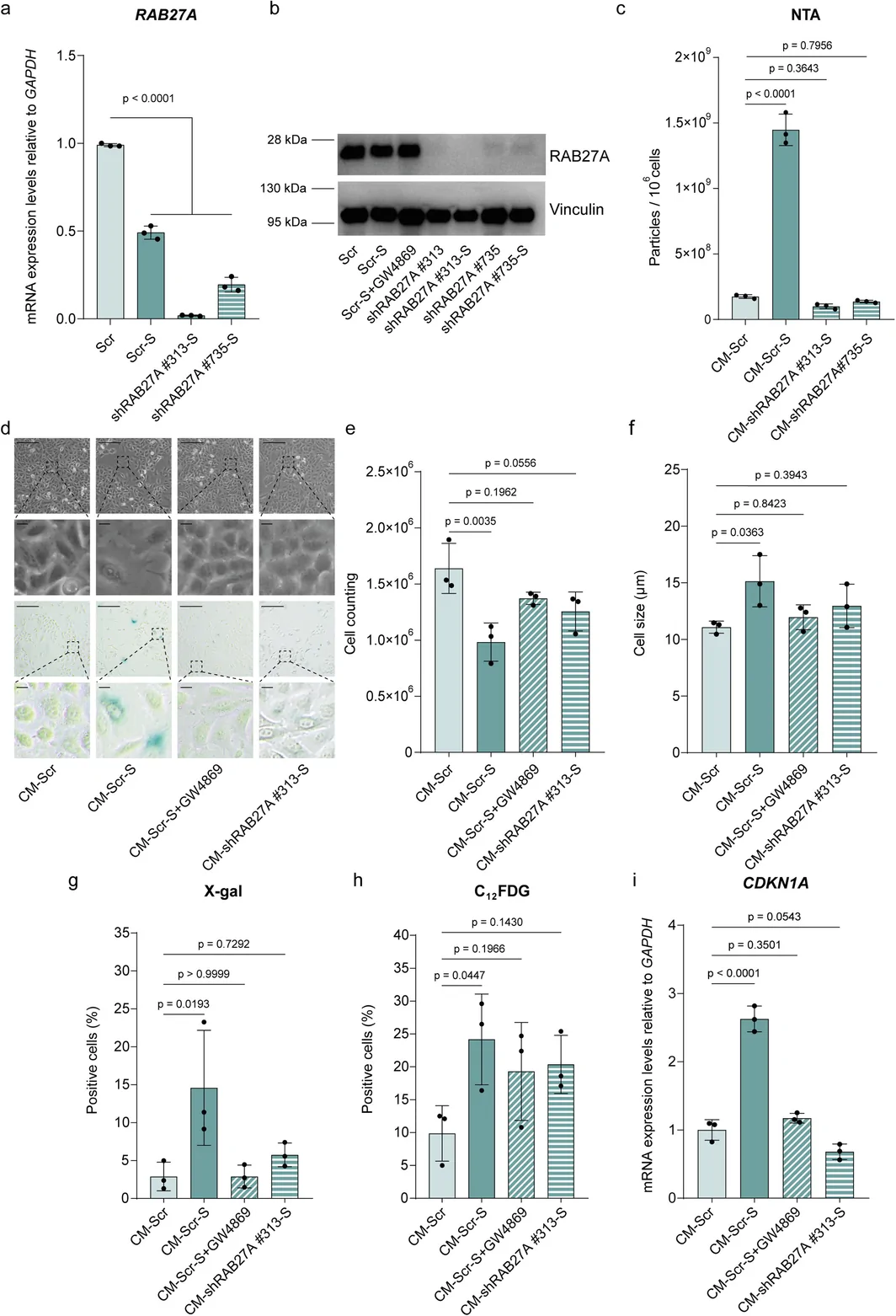
Genetic downregulation of RAB27A reduces EV secretion and paracrine senescence. (a) RT‐qPCR analysis of *RAB27A*. *GAPDH* was used as the housekeeping gene. (b) Western blot analysis of RAB27A. Vinculin was used as the loading control. (c) NTA analysis of particles in CM. (d) Representative images of morphology and SA‐β‐GAL staining using CMs from control (CM‐Scr), senescent (CM‐Scr‐S), GW4869‐treated senescent cells (CM‐Scr‐S + GW4869), and shRAB27A #313 senescent cells (CM‐shRAB27A #313‐S) (scale, 200 μm; scale in zoomed area, 20 μm). (e) Cell counting. (f) Cell size measurement. (g) Quantification of SA‐β‐GAL positive cells using X‐gal substrate. (h) Quantification of SA‐β‐GAL positive cells using C_12_FDG substrate. (i) RT‐qPCR analysis of *CDKN1A*. *GAPDH* was used as the housekeeping gene. Significance was calculated using one‐way ANOVA showing the exact *p*‐value. Less than 0.05 was considered statistically significant (a, c‐i, *n* = 3; b, *n* = 1).

To extend our results obtained with the chemical inhibitor GW4869, we took advantage of the genetic downregulation of *RAB27A* expression to reduce the release of EVs. RAB27A mediates the fusion between the multivesicular body (MVB) and the plasma membrane, and its downregulation is associated with a reduction in the amount of secreted EVs (Ostrowski et al. 2010). To target RAB27A we used two shRNAs: shRAB27A #313 and shRAB27A #735. First, we characterized the reduction of *RAB27A* expression achieved by each construct through RT‐qPCR and Western blot. While treatment with GW4869 had no effect on RAB27A levels, both shRNAs exhibited a robust capacity to reduce its expression, with shRAB27A #313 showing the strongest activity (Figure 3a,b). Importantly, neither #313 nor #735 shRNAs caused an alteration of the senescent phenotype of the cells as measured by SA‐β‐GAL staining using X‐gal (Figure S4a,b). In particular, we did not observe an alteration in the expression of several SASP factors after RAB27A knockdown (Figure S4c). To confirm the impact of *RAB27A* knockdown on the release of EVs, we measured the number of particles present in the different CMs by NTA (Figure 3c). While the CM from senescent cells expressing a non‐targeting shRNA (CM‐Scr‐S) showed a higher number of particles compared to the control (CM‐Scr), as expected, the senescent CM from cells expressing the two shRNAs targeting RAB27A (CM‐shRAB27A #313‐S and CM‐shRAB27A #735‐S) showed a clear reduction in the release of EVs (Figure 3c).

To analyze the effect of reducing the release of EVs by knocking down RAB27A, we added the CMs from control proliferating cells or after the expression of shRAB27A #313. Target cells incubated with CM‐Scr‐S reproduced the paracrine senescence induction already described, while both GW4869 treatment and RAB27A knockdown abolished this activity. Senescent cell morphology, reduction in cell numbers, SA‐β‐GAL activity, and an increase in *CDKN1A* expression only occurred when cells were incubated with CM‐Scr‐S but not with CM‐Scr‐S + GW4869 or CM‐shRAB27A #313‐S (Figure 3d–i and Figure S4d).

Altogether, our data indicate that the reduction of EVs secretion by genetic downregulation of *RAB27A* alleviates paracrine senescence.

### 2.4 Proteomic and Biophysical Characterization Reveals Distinct Features of Senescent EVs

**FIGURE 4:**
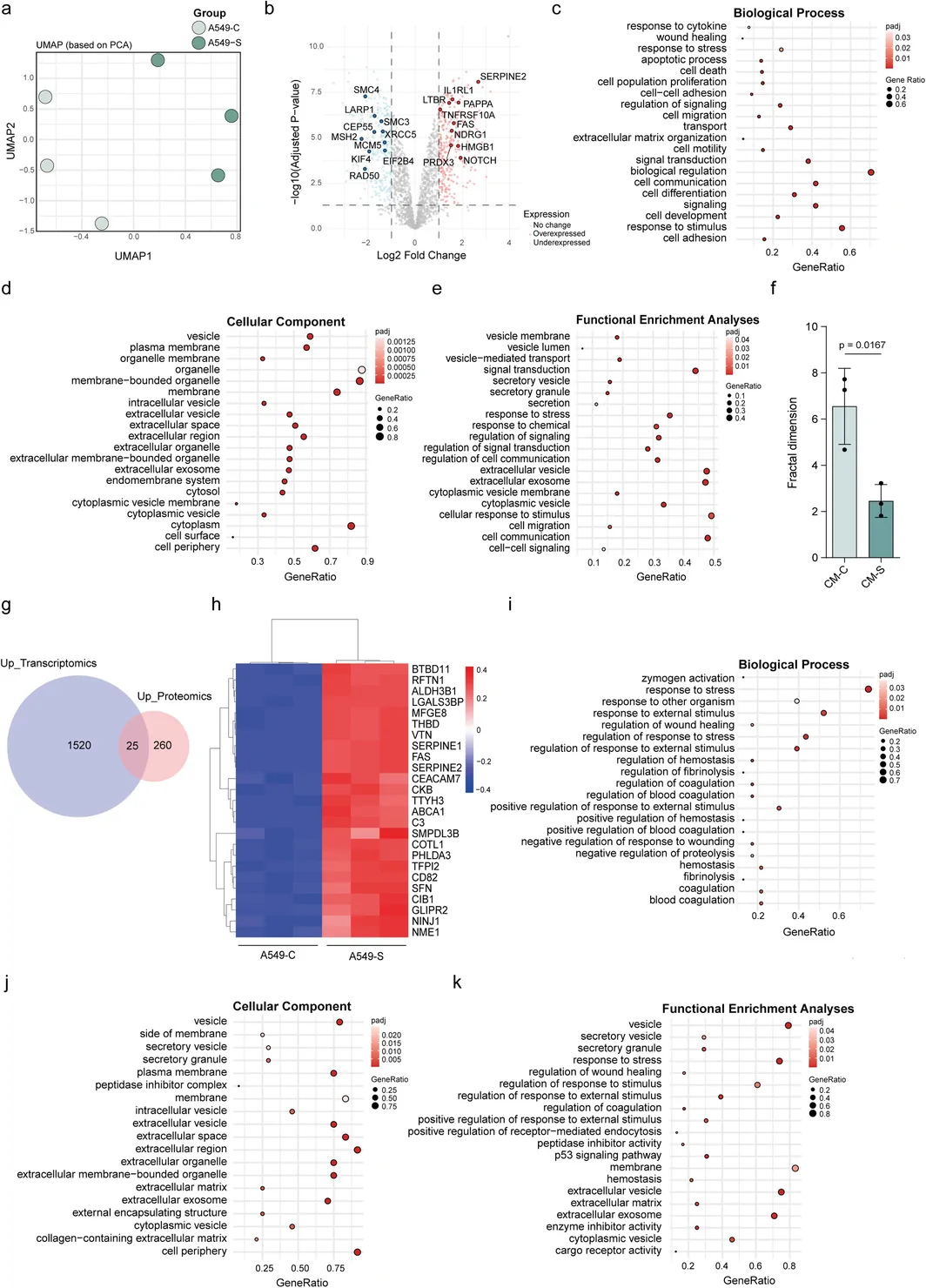
Senescent EVs exhibit a distinct proteomic profile. (a) UMAP showing group clustering of control (C‐EVs) and senescent (S‐EVs) EVs. (b) Volcano plot of differentially expressed proteins. (c) Dot plot analysis of differentially expressed Biological Process pathways, (d) Cellular Component terms, and (e) of Functional Enrichment Analyses pathways. (f) Fractal dimension of EVs analyzed by AF4 from control (CM‐C) or senescent (CM‐S) conditioned media. (g) Venn's diagram of upregulated transcripts (Up_transcriptomics) and proteins (Up_Proteomics) and their overlap. (h) Heatmap of overlapping proteins from Venn's diagram. (i) Dot plot analysis of differentially expressed Biological Process terms of overlapping proteins, (j) Cellular Component pathways, and (k) of Functional Enrichment Analyses pathways. Significance was calculated using one‐way ANOVA showing the exact *p*‐value. Less than 0.05 was considered statistically significant (f, *n* = 3).

To obtain a deeper understanding of the senescent EVs, we decided to perform a proteomic analysis of EVs isolated from control proliferating or senescent A549 cells (C‐EVs and S‐EVs, respectively). Samples from both groups separated clearly on a UMAP (Figure 4a). We managed to detect 1844 proteins; 285 of them were significantly upregulated in S‐EVs, and 267 were downregulated. We compared our proteomic profile with a previously described one obtained from EVs secreted by irradiation‐ or oncogene‐induced senescence, and found several common upregulated and downregulated proteins, as highlighted in the volcano plot (Figure 4b). The differential protein expression analysis by cellular component revealed terms related to extracellular vesicles and the extracellular space, validating the identity of our purified EVs. When we analyzed gene ontology terms related to biological process, we found several activities previously reported for senescent EVs, such as “wound healing”, “blood coagulation”, or “hemostasis” (Figure 4c–e) (Venturini et al. 2026).

To further characterize these vesicles, we next investigated their biophysical properties using asymmetrical flow field‐flow fractionation (AF4). CMs derived from senescent cells (CM‐S) contained particles exhibiting a marked decrease in their fractal dimension, indicative of a shift toward more compact and structurally homogeneous particles than the ones present in control CMs (CM‐C) (Figure 4f).

Next, we decided to combine the transcriptomic and proteomic analyses. We identified a subset of 25 differentially upregulated proteins that were also transcriptionally increased (Figure 4g,h). These 25 overlapping factors showed a strong upregulation at both the proteomic and transcriptomic levels and were again related to extracellular vesicles and the extracellular space, as well as biological processes related to wound healing and hemostasis (Figure 4i–k). In particular, some of the proteins and genes identified in our combined analysis (SERPINE1, SERPINE2, THBD, VTN) have been widely involved in wound healing, hemostasis, and blood coagulation (Sepúlveda et al. 2017; Sillen and Declerck 2021). Furthermore, some of these factors, such as SERPINE1, could also be potential effectors acting as proximal drivers of paracrine senescence (Jiang et al. 2017).

In summary, these results reveal that EVs isolated from bleomycin‐induced senescent A549 cells are defined not only by a distinct molecular cargo but also by a consistent remodeling of their biophysical properties.

### 2.5 Isolated Senescent EVs Can Independently Induce Paracrine Senescence

**FIGURE 5:**
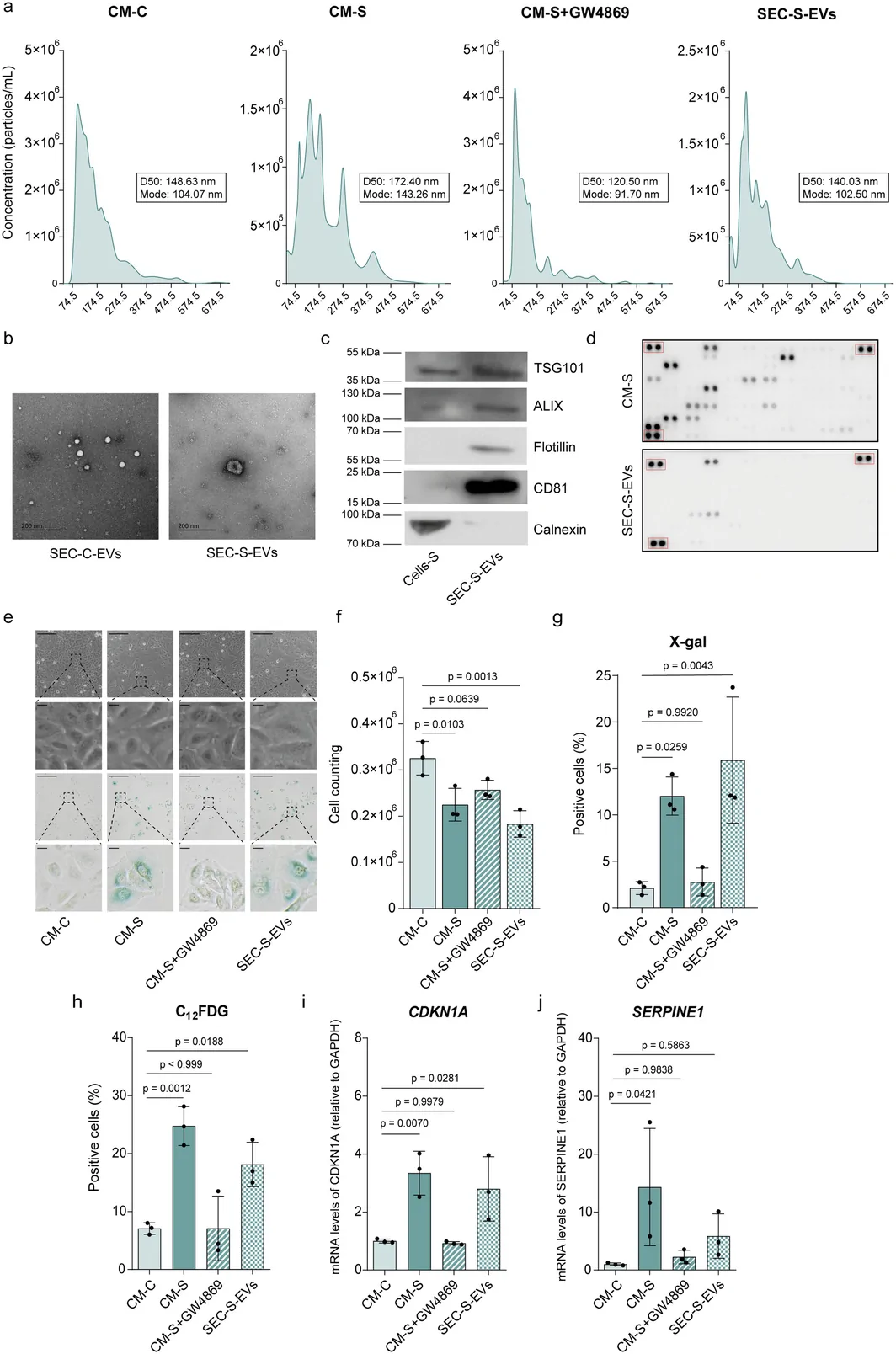
Isolated senescent EVs can independently induce paracrine senescence. (a) NTA analysis showing profiles, D50 and mode of particles within the CMs of control (CM‐C), senescent (CM‐S), senescent GW4869‐treated (CM‐S + GW4869) cells, and EVs isolated by SEC from senescent cells (SEC‐S‐EVs). (b) Transmission Electron Microscope (TEM) images of EVs isolated by SEC from control (SEC‐C‐EVs) and senescent (SEC‐S‐EVs) cells. (c) Western blot analysis showing the presence of canonical markers of EVs (TSG101, ALIX, Flotillin, and CD81) and absence of ER marker calnexin in EVs isolated from senescent (SEC‐S‐EVs) cells. (d) Cytokine array analysis of soluble proteins in CM‐S and SEC‐S‐EVs. (e) Representative images of morphology and SA‐β‐GAL staining of target cells treated with CMs from proliferative (CM‐C), senescent (CM‐S), GW4869‐treated senescent cells (CM‐S + GW4869), and EVs isolated by SEC from senescent cells (scale, 200 μm; scale in zoomed area, 20 μm). (f) Cell counting. (g) Quantification of SA‐β‐GAL positive cells using X‐gal substrate. (h) Quantification of SA‐β‐GAL positive cells using C_12_FDG substrate. (i) RT‐qPCR analysis of *CDKN1A*. *GAPDH* was used as a housekeeping gene. (j) RT‐qPCR analysis of *SERPINE1*. *GAPDH* was used as a housekeeping gene. Significance was calculated using one‐way ANOVA showing the exact *p*‐value. Less than 0.05 was considered statistically significant (a, e–j, *n* = 3; b–d, *n* = 1).

Our results showed that genetically or pharmacologically decreasing the number of EVs present in the CM from senescent cells abrogates the induction of paracrine senescence, suggesting that EVs may play an important role in this property of the SASP. To directly evaluate EV activity, we decided to isolate them from senescent CM by size‐exclusion chromatography (SEC). Purified EVs (SEC‐S‐EVs) were analyzed by NTA to verify if their size corresponds to the expected range for EVs. Compared to particles from CM samples (CM‐C, CM‐S and CM‐S‐GW4869), SEC‐S‐EVs showed a similar median hydrodynamic diameter distribution (D50) and mode (Figure 5a). Also, SEC‐S‐EVs were analyzed by transmission electron microscopy (TEM) and Western blot, showing that they express the canonical markers of EVs (TSG101, ALIX, Flotillin and CD81) and lack Calnexin, a well‐known marker of the endoplasmic reticulum, as expected (Welsh et al. 2024) (Figure 5b,c). To confirm that our EVs isolation method was efficiently removing soluble factors, we incubated cytokine arrays with our SEC‐S‐EVs preparation and compared the pattern of soluble factors with the one obtained by incubating with CM‐S. We observed a drastic reduction in the number and intensity of soluble factors detected by this method, with most of them undetectable in the SEC‐S‐EVs condition (Figure 5d). These data suggest that our preparations contain purified EVs, dissected from the soluble factors of the SASP.

To directly assess the potential activity of the EVs isolated from senescent cells, we added SEC‐S‐EVs to actively proliferating A549 cells and compared senescence induction with CM‐S. The treatment with CM‐S induced morphological changes resembling cell senescence and caused a reduction in cell proliferation as well as SA‐β‐GAL positive staining, while the pharmacological inhibition of EVs (CM‐S‐GW4869) reproducibly abrogated this effect. In contrast, the treatment with SEC‐S‐EVs recapitulated the same effects as the complete CM‐S (Figure 5e–j).

To further reinforce our findings, we performed a rescue experiment consisting of adding isolated senescent EVs to CM with reduced amounts of EVs after shRAB27A. Restoring the levels of senescent EVs rescued the lost paracrine senescence‐inducing activity of CM‐shRAB27A #313‐S (Figure S5a–e).

Taken together, these results reinforce the observation that the EVs from senescent cells can induce paracrine senescence independently of the rest of the components of the SASP.

## 3 Discussion

The senescence‐associated secretory phenotype is increasingly recognized as a multidimensional signaling system with soluble and vesicular arms that act in concert to remodel tissues and influence disease course (Basisty et al. 2020; Tanaka and Takahashi 2021; Wang et al. 2024). By integrating pharmacologic inhibition, genetic perturbation, unbiased proteomics, and functional reconstitution, our study delineates a central, nonredundant role for extracellular vesicles in transmitting senescence among tumor cells. Specifically, we show that reducing EV production pharmacologically or genetically attenuates the paracrine senescence activity of bleomycin‐induced senescent A549 cells without affecting the soluble SASP profile, while at the same time, purified senescent EVs are sufficient to drive paracrine senescence. Our data indicate that EVs are not merely correlates of the senescent state but active mediators of its propagation.

Prior work has established that soluble SASP components can induce senescence in neighboring cells (Acosta et al. 2013). However, the necessity and sufficiency of EVs have been less clear, especially in cancer contexts. Our experimental strategies converge on the same conclusion: depleting EVs curtails paracrine senescence, whereas purified senescent EVs restore it. The observation that GW4869 reduces EV number without altering producer‐cell senescence or soluble SASP composition strengthens the causal link to vesicular cargo rather than confounding changes in the global secretome, although we cannot rule out additional effects derived from the inhibitory activity of GW4869 over the N‐SMase that could be altering the lipid composition of the EVs or their cargo. Likewise, *RAB27A* knockdown, which diminishes EV release via impaired fusion of the multivesicular body with the plasma membrane, phenocopies the pharmacologic intervention, consolidating the mechanistic interpretation. Thus, our results show that EVs are necessary and sufficient mediators of paracrine senescence.

Proteomic characterization revealed that senescent EVs show a distinct proteomic profile. Senescent EVs harbor a signature enriched for extracellular vesicle/extracellular space components and biological processes such as wound healing, hemostasis, and tissue remodeling. This profile aligns with the broader concept that senescence can orchestrate microenvironmental changes conducive to both repair and pathology. The overlap between transcriptional upregulation and EV protein enrichment highlights candidate effectors whose biogenesis is coordinated at multiple regulatory layers. These factors may function as proximal drivers of the senescence response in recipient cells and serve as biomarkers of EV‐mediated signaling. However, at this stage we can only speculate about the possibility that some of these factors identified by proteomic and transcriptomic analyses are plausible effectors, since we have not experimentally validated them as drivers of the observed phenotype.

Therapy‐induced senescence (TIS) is a common outcome of genotoxic treatments and can paradoxically promote tumor relapse and resistance through persistent SASP signaling (Prasanna et al. 2021; Song et al. 2023; Wang et al. 2024). Our findings suggest that vesicular components of the SASP represent actionable levers to modulate TIS consequences. For instance, targeting EV biogenesis or release (e.g., via *RAB27A* pathways) could reduce the propagation of senescence to proliferating tumor cells and stromal elements, potentially curtailing the establishment of pro‐tumorigenic niches. Conversely, in settings where senescence reinforcement is desired, for example, in combination with immune‐mediated clearance, strategies to enhance or engineer EV cargo might be leveraged therapeutically.

In summary, our results define senescent EVs as necessary and sufficient effectors of paracrine senescence in tumor cells and unveil a distinct proteomic program associated with their activity. These insights refine the architecture of the SASP and provide a framework for targeting vesicular pathways to modulate senescence‐driven phenotypes in cancer.

Together, these results uncover a central role for EVs in mediating senescence propagation among tumor cells and provide mechanistic insight into how senescent cells influence their microenvironment. This work advances the understanding of SASP heterogeneity and identifies senescent EVs as key regulators of non–cell‐autonomous senescence responses with potential implications for cancer progression, therapeutic resistance, and senescence‐targeting interventions.

## 4 Methods

### 4.1 Cell Line Generation and Reagents

A549 (RRID:CVCL_0023) and MCF7 (RRID:CVCL_0031) cell lines were obtained from the ATCC. Both cell lines were cultured in high‐glucose DMEM (4500 mg/L, Sigma‐Aldrich) supplemented with 10% FBS (Sigma‐Aldrich), 1% penicillin–streptomycin (Sigma‐Aldrich), 1% L‐glutamine (Sigma‐Aldrich), and maintained in an incubator at 37°C and 5% CO_2_ atmosphere. Cells were routinely tested for mycoplasma. Non‐targeting scramble plasmid (Scr), shRAB27A #313 and shRAB27A #735 were a kind gift from Dr. Alberto Fraile‐Ramos (Fraile‐Ramos et al. 2010). For lentiviral transduction, 5 × 10^6^ HEK‐293 T cells were seeded 24 h before transfection with a plasmid mixture in the presence of polyethyleneimine reagent 1 mg/mL (PEI, Polysciences) in a 1:1 plasmid DNA: PEI proportion. Third‐generation lentiviral packaging plasmids pLP1, pLP2 and pLP‐VSVG (ViraPower Lentiviral Packaging Mix, Invitrogen) were used. 36 h post‐transfection, we collected lentiviral‐enriched conditioned media every 12 h to treat target A549 cells for three consecutive rounds. The conditioned media were filtered through a 0.45 μm filter and supplemented with 8 μg/mL of polybrene reagent (Sigma‐Aldrich). We treated A549 cell line with these conditioned media and selected with puromycin (Millipore) at 1 μg/mL.

For chemotherapy‐induced senescence we used bleomycin (Mylan pharmaceutics) at 20 μM for 5 days. After senescence induction, conditioned media were collected following 24 h of incubation with complete DMEM. Conditioned media were centrifuged for 10 min at 500 g to deplete cellular debris and apoptotic bodies and stored at 4°C until use.

For irradiation‐induced senescence, cells were exposed to X‐ray irradiation using a CLINAC 2300 IX linear accelerator (Varian Medical Systems, CLINAC 2300 IX) at a total dose of 20 Gy. Medium was refreshed 2 h post‐irradiation and cells were cultured for an additional 5 days to fully become senescent.

For GW4869 treatment, cells were treated at 10 μM for 24 h following senescence induction. Then, cells were washed with 1× PBS and media were conditioned for 24 h.

### 4.2 RT‐qPCR

For gene expression analysis, we extracted total RNA from cell cultures using the Nucleospin RNA Kit (Macherey‐Nagel) following the manufacturer's instructions. Then, we converted RNA into cDNA using the High‐Capacity cDNA Reverse Transcription Kit (Applied Biosystems). For each reaction, we used 33 ng of cDNA, oligonucleotides at a 0.25 μM final concentration, 5 μL NZYSpeedy qPCR Green Master Mix (2X), ROX (NZYTech), and nuclease‐free water up to 10 μL total volume. Quantification was performed in the AriaMX Real‐Time PCR System (Agilent Technologies). Primer sequences were:

CDKN1A: 5′‐CCTGTAACTGTCTTGTACCCT‐3′, 5′‐GCGTTTGGAGTGGTAGAAATCT‐3′.

SERPINE1: 5′‐CTCATCAGCCACTGGAAAGGCA‐3′, 5′‐GACTCGTGAAGTCAGCCTGAAAC‐3′.

RAB27A: 5′‐GCTTTGGGAGACTCTGGTGTA‐3′, 5′‐TCAATGCCCACTGTTGTGATAAA‐3′.

GAPDH:5′‐TCCATGACAACTTTGGCATCGTGG‐3′, 5′‐GTTGCTGTTGAAGTCACAGGAGAC‐3′.

CCL2: 5′‐AGCTCGCACTCTCGCCTCCAG‐3′, 5′‐GGCATTGATTGCATCTGGCTGAGC‐3′.

GDF15: 5′‐GACCCTCAGAGTTGCACTCC‐3′, 5′‐GCCTGGTTAGCAGGTCCTC‐3′.

PDGFA: 5′‐CAGCGACTCCTGGAGATAGACT‐3′, 5′‐CGATGCTTCTCTTCCTCCGAATG‐3′.

CXCL1: 5′‐GAAAGCTTGCCTCAATCCTG‐3′, 5′‐CACCAGTGAGCTTCCTCCTC‐3′.

VEGFA: 5′‐TACCTCCACCATGCCAAGTG‐3′, 5′‐GATGATTCTGCCCTCCTCCTT‐3′.

### 4.3 EVs Isolation

For EVs isolation, we followed the instructions of Smart SEC Single for EV Isolation kit (System Biosciences). For functional analysis, we resuspended EVs preparations in DMEM supplemented with 40% FBS, 4% penicillin/streptomycin, and 4% L‐glutamine in a 3:1 proportion to reconstitute the standard complete media.

### 4.4 NTA Characterization

To determine particle concentration, conditioned media was analyzed by nanoparticle tracking analysis (NTA). For direct conditioned media analysis, samples were diluted 1:10 in 1× PBS, whereas isolated EVs were diluted 1:100 in 1× PBS. Measurement was performed using Nanosight NTA v2.3 (Malvern Instruments). We used a detection threshold of 11 and a camera level of 15.

### 4.5 Cell Morphology and Proliferation Analysis

For cell counting and size, cells were trypsinized, resuspended in DMEM supplemented with 10% FBS, and analyzed by automatic cell counter Luna II (Logos Biosystems). Cell morphology images were taken in an Axio Vert.A1 Microscope (Zeiss).

### 4.6 Cellular Staining

For clonogenic assays, cells were seeded at low confluency and allowed to proliferate for 14 days. Then, cells were fixed with 4% paraformaldehyde (Electron Microscopy Sciences) and stained with 0.05% crystal violet solution (Sigma) for 30 min. After staining, cells were washed and crystal violet was eluted with 10% acetic acid. Optical density was measured at 570 nm using Epoch2 microplate reader (Biotek).

For SA‐β‐GAL activity detection, we used the chromogenic X‐gal substrate. Cell culture was washed with 1× PBS and fixed with 2% paraformaldehyde (Electron Microscopy Sciences) and 0.2% glutaraldehyde for 15 min at room temperature. After fixation, cells were washed with 1× PBS and stained with X‐gal solution (X‐gal solution was prepared by mixing citric acid/sodium phosphate (pH 6.0) at 40 mM, NaCl at 150 mM, K_3_Fe(CN)_6_ at 5 mM, K_4_Fe(CN)_6_ at 5 mM, MgCl_2_ at 2 mM, and X‐gal (5‐bromo‐4‐chloro‐3‐indolyl β‐D‐galactose) at 1 mg/mL in distilled water) at 37°C overnight. Cells were washed at the end of the staining with 1× PBS. Images were taken using an Axio Vert.A1 Microscope (Zeiss). To quantify positive cells, 10 images per condition were analyzed.

For flow cytometry analysis of senescence, C_12_FDG substrate was used according to the instructions of the CellEvent Senescence Green Flow Cytometry Assay kit (Invitrogen). Samples were analyzed on a Cytoflex Flow Cytometer (Beckman Coulter), with 10,000 events recorded per sample and using FlowJo v10 software.

EdU incorporation assays were performed using Click‐iT EdU Alexa Fluor 488 Imaging (Invitrogen) according to the manufacturer's instructions. Nuclei were counterstained with DAPI. A549 cells were incubated with 10 μM EdU for 2 h at 37°C. Images were taken with Axio Vert.A1 Microscope (Zeiss) and 10 images per condition were quantified.

### 4.7 Western Blot

Cellular lysates were prepared in 1× RIPA buffer containing protease and phosphatase inhibitor cocktails (4693159001 and 4906845001, Sigma‐Aldrich). To measure protein concentration, we used DC Protein Assay (BIO‐RAD), following the manufacturer's protocol.

A total of 25 μg of protein per sample was electrophoresed in 12% precast polyacrylamide gels (Invitrogen) using the XCell SureLock (Invitrogen) system and transferred to a 0.45 μm PVDF membrane (Merck‐Millipore). Membranes were blocked with 5% milk solution and incubated with primary antibodies for GAPDH (sc‐32233, Santa Cruz Biotechnology 1:1000), β‐Actin (sc‐8432, Santa Cruz Biotechnology, 1:1000), Vinculin (sc‐76,314, Santa Cruz Biotechnology 1:2000), RAB27A (#69295, Cell Signaling Technology, 1:1000), p53 (sc‐6243, Santa Cruz Biotechnology, 1:1000), and p21 (#2947, Cell Signaling Technology, 1:1000) overnight at 4°C. Then, membranes were incubated with HRP‐conjugated secondary antibodies anti‐mouse IgG‐HRP (#31430, Invitrogen, 1:10000) and anti‐rabbit IgG‐HRP (#31460, Invitrogen, 1:10000) for 1 h. Chemiluminescent detection was performed using the SuperSignal West Pico PLUS substrate (Thermo Scientific) in a ChemiDoc MP Imaging System (BIO‐RAD).

To analyze EVs protein levels, EVs were lysed separately using 2% SDS. Protein concentration was determined using the Micro BCA Protein Assay Kit (Thermo Scientific). A total of 25 μg of total protein was separated into 12% precast polyacrylamide gels for electrophoresis. After transferring the proteins into a PVDF membrane, the blot was blocked with a 5% milk solution. Membranes were incubated overnight at 4°C with the following primary antibodies: Alix (ab88743, Abcam, 1:500), Calnexin (Ab22595, Abcam, 1:1000), TSG101 (SC‐7964, Santa Cruz Biotechnology, 1:1000), Flot‐1 (SC74566, Santa Cruz Biotechnology, 1:100), and CD81 (SC166029, Santa Cruz Biotechnology, 1:500). Afterward, membranes were incubated for 1 h with HRP‐conjugated secondary antibodies anti‐mouse IgG‐HRP (#31430, Invitrogen, 1:10000) and anti‐rabbit IgG‐HRP (#31460, Invitrogen, 1:10000). Chemiluminescent detection was performed using the SuperSignal West Pico PLUS substrate (Thermo Scientific), and signals were visualized using radiographic X‐ray films (FUJI).

### 4.8 Cytokine Array

Soluble factors released by senescent cells were analyzed by cytokine array following the instructions of the Proteome Profiler Human XL Cytokine Array Kit (Bio‐Techne). Arrays were revealed using a ChemiDoc MP Imaging System (BIO‐RAD).

### 4.9 Tem

Copper grids coated with a collodion/carbon film were subjected to an electrical discharge for 15 s to enhance sample adhesion to the grid. Grids were incubated with the sample for 5 min at room temperature. Excess liquid was removed by blotting, and grids were washed with 50 μL of distilled water for 10 s. After a second blotting step, grids were stained with 2% uranyl acetate for 30 s.

### 4.10 AF4 Separation

Asymmetric flow field‐flow fractionation (AF4) was performed on a Postnova AF2000 system equipped with a degasser, tip and focus pumps, an autosampler, and a Waters Fraction Collector III. The separation channel contained a metallic frit, a 500 μm spacer, and a regenerated cellulose (RC) membrane with a 10 kDa molecular weight cutoff (Postnova, Z AF4 MEM 612 10kD). Detection was achieved using UV absorbance at 280 nm and multi angle light scattering (MALS). CMs (52 μL per injection) from proliferative and senescent cells were pre purified using Amicon Ultra centrifugal filters (100 kDa MWCO, RC membrane; Merck Millipore). The channel flow was set to 0.5 mL/min. Cross flow started at 1.2 mL/min and decreased linearly to 0 over 25 min, followed by an elution phase at constant cross flow = 0. EVs eluted after free proteins (Rg = 5–10 nm). The region of interest corresponding to EVs exhibited an Rg of ~60–70 nm, consistent with literature values. The carrier liquid was PBS supplemented with 0.2 mM sodium azide (Sigma Aldrich, 71289). For the focusing step, flow rates were set to 1.5 mL/min (focus pump), 0.2 mL/min (injection flow), and 1.2 mL/min (cross flow), yielding a channel flow of 0.5 mL/min. A 3 min injection time ensured that the full sample volume reached the focus point and produced a stable and effective focusing zone.

### 4.11 RNA Seq Library Preparation, Sequencing and Generation of FastQ Files

Total RNA (100 ng) was used to generate barcoded RNA‐seq libraries using the NEBNext Ultra II Directional RNA Library preparation kit (New England Biolabs) according to manufacturer's instructions. First, poly A+ RNA was purified using poly‐T oligo‐attached magnetic beads followed by fragmentation and first and second cDNA strand synthesis. Next, cDNA ends were repaired and adenylated. The NEBNext adaptor was then ligated followed by second strand removal, uracile excision from the adaptor and PCR amplification. The size of the libraries was checked using the Agilent 2100 Bioanalyzer and the concentration was determined using the Qubit fluorometer (ThermoFisher).

Libraries were sequenced on a HiSeq4000 (Illumina) to generate 60 bases single reads.

FastQ files for each sample were obtained using bcl2fastq 2.20 Software (Illumina).

### 4.12 Proteomic Analysis

EVs' protein samples were obtained from FBS‐free conditioned media. Samples were solubilized with Tris–HCl 100 mM, pH 8.0, 8 M urea solution and 10 μg of protein were digested with standard ASP1 protocol. Digested samples were processed by mass spectrometry (LC–MS/MS) through Ultimate 3000 RSLCnano (Dionex) system coupled with mass spectrometer Exactive HF‐X (ThermoScientific). Data was processed with MaxQuantum (v 1.6.1.10) using standard configurations. We crossed our data with UniprotKB/Swiss‐Prot, July 2018, 20,373 sequences database. Label‐free quantification was performed using the “match between runs” algorithm (match window of 0.7 min and alignment window of 20 min). The minimum peptide length was set to 7 amino acids, and a maximum of 2 missed tryptic cleavages was allowed. Results were filtered at a false discovery rate (FDR) of 1% at both the peptide and protein levels. Then proteinGroups.txt file was imported into Prostar (v1.14.7) for further statistical analysis. For the “Senescent versus Control” comparison, proteins with an absolute log2 fold change > 1 and an FDR‐adjusted *p*‐value < 0.05 (Limma) were considered differentially regulated.

### 4.13 Exploratory Data Analysis (EDA) and Functional Enrichment Tools

Raw normalized abundance values were imported from Excel files into R (v4.4.2). Missing values were imputed using a distribution‐based approach prior to downstream analyses. Row‐wise *z*‐scores were calculated for visualization, and heatmaps were generated with hierarchical clustering (Euclidean distance, Ward's method).

Principal Component Analysis (PCA) was performed on the variance‐stabilized and transposed abundance matrix, and the top principal components were used to assess variation among experimental conditions. UMAP was applied to the leading principal components to visualize sample clustering in two dimensions.

Gene Set Enrichment Analysis (GSEA) was performed using ranked protein lists and curated gene sets from KEGG, Reactome, and senescence‐related signatures. Significantly overexpressed proteins were further analyzed using the g:Profiler web tool for GO, Reactome, and KEGG enrichment. Results were visualized in R and overlap with the SASPAtlas database was assessed to identify known senescence‐associated secretory phenotype (SASP) components.

### 4.14 Statistical Analysis

All data are represented as mean ± standard deviation. Statistical significance between two groups was calculated using Student's *t*‐test. For comparisons involving more than two groups, we used one‐way ANOVA with Tukey's post hoc test. Statistical significance was represented as the exact *p*‐value, considering less than 0.05 as statistically significant.

For differential expression analysis, log2 fold changes were calculated from group means and statistical significance was assessed using *t*‐tests with Benjamini‐Hochberg correction for multiple testing (FDR). Proteins with |log2FC| > 1 and adjusted *p*‐value < 0.05 were considered significantly differentially expressed. Volcano plots were used for visualization.

Figure plots were generated with Graphpad Prism 9.4.1.

## Author Contributions

V.E.‐S., A.M.‐D. and S.D.S.‐A. performed most of the experimental work; P.P., P.L.‐F., M.A.P., A.F.‐F., R.P.‐P., R.L.‐B. and M.G.‐B. helped with experiments and reagents; J.R.‐O., M.J.A., M.M., and E.P. helped with the analysis of EVs; G.N.C. and C.S.M. helped with TEM; P.X.‐E. and J.M. performed proteomics; R.A.‐V., E.V.D.L. and A.D. performed transcriptomics; V.E.‐S., M.C., and S.D.S.‐A. wrote the manuscript; M.C., and S.D.S.‐A. conceptualized and supervised the project.

## Funding

This work was supported by Ministerio de Ciencia, Innovación y Universidades, PID2021‐125479OB‐I00, PID2024‐159335OB‐I00, FPU23/02493, FPU24/00846, CEX2024‐001463‐M, JDC2022‐049032‐I. Axencia Galega de Innovación, IN607B2024/13, ED481A 2022/432, ED481A‐2025‐101, ED431G/2023/02. Fundacion AECC, PRDLC246226FAIL.

## Conflicts of Interest

The authors declare no conflicts of interest.

## Supporting information


**Figure S1:** Characterization of chemotherapy‐induced cellular senescence in A549 cells. (a) Cell size measurement of proliferative (A549‐C) and senescent (A549‐S) cells. (b) Flow cytometry plots and quantification of SA‐β‐GAL positive cells using C_12_FDG substrate. (c) RT‐qPCR analysis of *CDKN1A*. *GAPDH* was used as housekeeping gene. (d) Western blot analysis of senescence markers (p53 and p21). GAPDH and Actin were used as loading controls. (e) Clonogenicity assay and quantification. (f) EdU incorporation assay and quantification (scale, 200 μm). (g) GSEA plots of the Reactome_Cell_Cycle and Fridman_Senescence_up gene sets. (h) Heatmap and list of differentially expressed genes. (i) Dot plot analysis of differentially expressed genes analyzed using the Reactome pathways and KEGG databases. (j) Dot plot analysis of differentially expressed genes using the Biological process terms in Gene Ontology. (k) Flow cytometry plots of cells treated with control (CM‐C) and senescent (CM‐S) conditioned media. Significance was calculated using Student's *t*‐test showing the exact *p*‐value. Less than 0.05 was considered statistically significant (*n* = 3, except d, *n* = 1).
**Figure S2:** EVs derived from irradiation‐induced senescent A549 and SK‐MEL‐147 cells induce paracrine senescence. (a) Representative images of cell morphology and SA‐β‐GAL staining of CM producer cells, scale represents 100 μm and zoomed area scale represents 10 μm. (b) Quantification of SA‐β‐GAL positive cells using X‐gal substrate. (c) Clonogenicity assay (d) Representative images of cell morphology and SA‐β‐GAL staining of target cells, scale represents 100 μm and zoomed area scale represents 10 μm. (e) Cell size measurement. (f) Cell counting. (g) Quantification of SA‐β‐GAL positive cells using X‐gal substrate. (h) RT‐qPCR analysis of *CDKN1A. GAPDH* was used as housekeeping gene. (i) RT‐qPCR analysis of *SERPINE1. GAPDH* was used as housekeeping gene (*n* = 1).
**Figure S3:** GW4869‐treated senescent cells retain senescence phenotype. (a) Representative images of cell morphology and SA‐β‐GAL staining (scale, 200 μm; scale in zoomed area, 20 μm). (b) Cell size measurement. (c) Quantification of SA‐β‐GAL positive cells using X‐gal substrate. (d) Flow cytometry plots and quantification of SA‐β‐GAL positive cells using C_12_FDG substrate. (e) Flow cytometry plots of cells treated with conditioned media derived from control (CM‐C), senescent (CM‐S) and GW4869‐treated senescent cells (CM‐S + GW4869). Significance was calculated using one‐way ANOVA showing the exact *p*‐value. Less than 0.05 was considered statistically significant (*n* = 3).
**Figure S4:** Senescence phenotype remains unaltered after RAB27A knockdown. (a) Representative images of morphology and SA‐β‐GAL staining (scale, 200 μm; scale in zoomed area, 20 μm). (b) Quantification of SA‐β‐GAL positive cells using X‐gal substrate. (c) RT‐qPCR analysis of *CDKN1A, SERPINE1, CCL2, GDF15, PDGFA, CXCL1* and *VEGFA*. *GAPDH* was used as housekeeping gene. (d) Flow cytometry plots of cells treated with conditioned media derived from control (CM‐Scr), senescent (CM‐Scr‐S), or GW4869‐treated senescent (CM‐Scr‐S + GW4869) cells, all of them expressing control Scrambled shRNA, and from RAB27A shRNA knockdown senescent cells (CM‐shRAB27A #313‐S). Significance was calculated using one‐way ANOVA. Exact *p*‐values are shown. Less than 0.05 was considered statistically significant (*n* = 3 except c, *n* = 1).
**Figure S5:** Supplementation of senescent EVs restores the paracrine senescence activity. (a) Representative images of morphology and SA‐β‐GAL staining, scale represents 100 μm and zoomed area scale represents 10 μm. (b) Cell size measurement. (c) Cell counting. (d) Quantification of SA‐β‐GAL positive cells using X‐gal substrate. (e) Quantification of SA‐β‐GAL positive cells using C12FDG substrate (*n* = 1).

## Data Availability

The data that support the findings of this study are available on request from the corresponding author. The data are not publicly available due to privacy or ethical restrictions.
