# Supplementary material for: Extracellular Vesicles From Senescent Cancer Cells Are Necessary and Sufficient to Drive Paracrine Senescence
Source: Aging Cell. 2026 Sep 23;25(10):e70722. doi: 10.1111/acel.70722 (PMC13598935; doi:10.1111/acel.70722)
Supplement: Supplementary file 1 [file ACEL-25-e70722-s001.docx]

**SUPPLEMENTARY FIGURES**

**Extracellular Vesicles from Senescent Cancer Cells Are Necessary and Sufficient to Drive Paracrine Senescence**

Valentin Estevez-Souto^1^, Alex Miralles-Dominguez^1^, Pablo Pedrosa^1^, Patricia Lado-Fernandez^1^, Miguel A. Prados^1^, Alejandro Failde-Fiestras^1^, Raquel Paredes-Paredes^1^, Jorge Ruz-Ortega^2^, Maria J. Alonso^2,3,4^, Martina Migliavacca^5^, Ester Polo^6^, Rebeca Alvarez-Velez^7^, Enrique Vazquez de Luis^7^, Ana Dopazo^7^, Gabriela N. Condezo^8^, Carmen San Martin^8^, Miguel Gonzalez-Barcia^9,10^, Ramón Lobato-Busto^11^, Pilar Ximénez-Embún^12^, Javier Muñoz^12,13,14^, Manuel Collado^1,15*^, Sabela Da Silva-Alvarez^1,6*^

*Running title: Paracrine senescence activity of extracellular vesicles*

**Supplementary Fig. 1. Characterization of chemotherapy-induced cellular senescence in A549 cells.** (a) Cell size measurement of proliferative (A549-C) and senescent (A549-S) cells**.** (b) Flow cytometry plots and quantification of SA-β-GAL positive cells using C_12_FDG substrate. (c) RT-qPCR analysis of *CDKN1A*. *GAPDH* was used as housekeeping gene. (d) Western blot analysis of senescence markers (p53 and p21). GAPDH and Actin were used as loading controls. (e) Clonogenicity assay and quantification**.** (f) EdU incorporation assay and quantification (scale, 200 μm)**.** (g) GSEA plots of the Reactome_Cell_Cycle and Fridman_Senescence_up gene sets**.** (h) Heatmap and list of differentially expressed genes. (i) Dot plot analysis of differentially expressed genes analyzed using the Reactome pathways and KEGG databases. (j) Dot plot analysis of differentially expressed genes using the Biological process terms in Gene Ontology. (k) Flow cytometry plots of cells treated with control (CM-C) and senescent (CM-S) conditioned media. Significance was calculated using Student’s t-test showing the exact p-value. Less than 0.05 was considered statistically significant (n=3, except d, n=1).

**Supplementary Fig. 2. EVs derived from irradiation-induced senescent A549 and SK-MEL-147 cells induce paracrine senescence.** (a) Representative images of cell morphology and SA-β-GAL staining of CM producer cells, scale represents 100 μm and zoomed area scale represents 10 μm. (b) Quantification of SA-β-GAL positive cells using X-gal substrate**. (c)** Clonogenicity assay (d) Representative images of cell morphology and SA-β-GAL staining of target cells, scale represents 100 μm and zoomed area scale represents 10 μm. (e) Cell size measurement. (f) Cell counting. (g) Quantification of SA-β-GAL positive cells using X-gal substrate. (h) RT-qPCR analysis of *CDKN1A. GAPDH* was used as housekeeping gene. (i) RT-qPCR analysis of *SERPINE1. GAPDH* was used as housekeeping gene. (n=1)

**Supplementary Fig. 3. GW4869-treated senescent cells retain senescence phenotype.** (a) Representative images of cell morphology and SA-β-GAL staining (scale, 200 μm; scale in zoomed area, 20 μm)**.** (b) Cell size measurement**.** (c) Quantification of SA-β-GAL positive cells using X-gal substrate**.** (d) Flow cytometry plots and quantification of SA-β-GAL positive cells using C_12_FDG substrate**.** (e) Flow cytometry plots of cells treated with conditioned media derived from control (CM-C), senescent (CM-S) and GW4869-treated senescent cells (CM-S+GW4869). Significance was calculated using one-way ANOVA showing the exact p-value. Less than 0.05 was considered statistically significant (n=3).

**Supplementary Fig. 4. Senescence phenotype remains unaltered after RAB27A knockdown.** (a) Representative images of morphology and SA-β-GAL staining (scale, 200 μm; scale in zoomed area, 20 μm)**.** (b) Quantification of SA-β-GAL positive cells using X-gal substrate**.** (c) RT-qPCR analysis of *CDKN1A, SERPINE1, CCL2, GDF15, PDGFA, CXCL1* and *VEGFA*. *GAPDH* was used as housekeeping gene. (d) Flow cytometry plots of cells treated with conditioned media derived from control (CM-Scr), senescent (CM-Scr-S), or GW4869-treated senescent (CM-Scr-S+GW4869) cells, all of them expressing control Scrambled shRNA, and from RAB27A shRNA knockdown senescent cells (CM-shRAB27A #313-S). Significance was calculated using one-way ANOVA. Exact p-values are shown. Less than 0.05 was considered statistically significant (n=3 except c, n=1).

**Supplementary Fig. 5. Supplementation of senescent EVs restores the paracrine senescence activity.** (a) Representative images of morphology and SA-β-GAL staining, scale represents 100 μm and zoomed area scale represents 10 μm. (b) Cell size measurement. (c) Cell counting. (d) Quantification of SA-β-GAL positive cells using X-gal substrate. (e) Quantification of SA-β-GAL positive cells using C12FDG substrate. (n=1).
